# Spatial Analysis of Urban Form and Pedestrian Exposure to Traffic Noise

**DOI:** 10.3390/ijerph8061977

**Published:** 2011-06-03

**Authors:** Ni Sheng, U. Wa Tang

**Affiliations:** 1 Faculty of Management and Administration, Macau University of Science and Technology, Avenida Wai Long, Taipa, Macau, China; E-Mail: nsheng.1@gmail.com; 2 Department of Civil and Environmental Engineering, University of Macau, Choi Kai Yau Building, Taipa, Macau, China

**Keywords:** urban form, traffic volume, traffic noise, GIS

## Abstract

In the Macao Peninsula, the high population density (49,763 inhabitants/km^2^) and the lack of control over the number of vehicles (460 vehicles/km) have led to an increase in urban pollution. To provide useful information to local government and urban planners, this paper investigates the spatial distribution of traffic noise in the Macao Peninsula. The interactions among urban form, traffic flow and traffic noise are addressed. Considering the spatial nature of urban geometry and traffic, a high-resolution GIS-based traffic noise model system is applied. Results indicate that the Macao Peninsula has fallen into a situation of serious traffic noise pollution. About 60% of traffic noise levels along the major pedestrian sidewalks in the evening peak hour exceed the National Standard of 70 dB(A) in China. In particular, about 21% of traffic noise levels along the pedestrian sidewalks are above the National Standard by 5 dB(A). Noticeably, the high pedestrian exposure to traffic noise in the historical urban area reduces the comfort of tourists walking in the historic centre and is ruining the reputation of the area as a World Cultural Heritage site.

## 1. Introduction

Traffic noise is an important environmental health problem affecting the health and wellbeing of the people exposed. Excessive exposure to noise might be considered a health risk in that noise may contribute to the development and aggravation of stress related conditions such as high blood pressure, coronary disease, ulcers, colitis, and migraine headaches [1].

Many traffic noise prediction models have been designed for traffic noise assessment. In traffic noise modeling, the noise level at a receptor position due to traffic emission source is modeled as a function of the traffic conditions (*i.e.*, traffic volume, traffic composition, traffic speed), road gradient, road surface nature, absorbent ground cover percentage, street configuration, and distance between the traffic emission source and the receptor. The traffic emission source can be considered as point or line source. Traffic noise models assuming point emission source include the United States Federal Highway Administration Traffic Noise Model (FHWA-TNM) [2] and the model by the Acoustical Society of Japan (ASJ) [3]. While those assuming line emission source include the Calculation of Road Traffic Noise (CRTN) model in the United Kingdom [4], the RLS90 model in Germany [5], the MITRA model in France [6], and the STL-86 model in Switzerland [7].

Traffic noise modeling in urban areas with complex traffic conditions and urban geometries requires processing a large amount of complex geographically referenced data such as street configuration, road gradient, road surface nature, and emission sources. Therefore, it has a strong desire to integrate traffic noise models with a Geographic Information System (GIS) that enables capture, modeling, manipulation, retrieval, analysis, and presentation of geographically referenced data. The GIS is an important tool in spatial analysis and modeling. A number of researchers have used the integrated GIS traffic noise prediction models to estimate the level of noise in urban areas [8–16].

The importance of urban form on sustainable development has been recognized in recent years. Urban land-use pattern and urban geometry are two basic characteristics of urban form. They fundamentally determine transportation demands, which directly affect noise and air pollution. Some researchers have studied the spatial relationship among urban form, traffic volume and traffic noise [17–26]. In the Macao Peninsula, the urban form is highly compact due to the high population density (49,763 inhabitants/km^2^) and mixed-used development (*i.e.*, a mixture of residential, commercial, industrial, or other land uses in a building or set of buildings). It is therefore crucial to investigate the influences of the existing compact urban forms on noise pollution.

This paper applies a GIS-based traffic noise model system to investigate the influences of existing urban forms on vehicle transport and pedestrian exposure to traffic noise in the Macao Peninsula. The model system has been developed and verified in our previous work [27] and a brief description is given in Section 2. The spatial distribution of traffic noise modelled at 1955 receptor points along the pedestrian sidewalks in the Macao Peninsula is presented in Section 3. The spatial relationships among urban form, traffic flow and traffic noise are also discussed. Finally the conclusions are given in Section 4.

## 2. GIS-Based Traffic Noise Model System

Considering the spatial nature of urban structure and noise distribution, the authors have developed a GIS-based traffic noise model system with a high spatial resolution down to individual buildings along both sides of the street. The system integrates the road traffic noise model CRTN [4], digital maps, administrative databases, an urban landscape model and the GIS. The model system has been verified in our previous work [27]. To the convenience of the reader, a brief description of the model system is given below. Interested readers could consult the paper [27] for more details.

The traffic noise levels along the pedestrian sidewalks in the Macao Peninsula are calculated using the CRTN model available in the Department of Transport’s Technical Memorandum in the United Kingdom [4]. The CRTN model is among the first systematic scheme developed to predict noise levels due to road traffic. It has been used in Hong Kong, U.K. and New Zealand for the assessment of road traffic environmental impacts and found to be a good representation of actual measured noise levels.

The calculation of the CRTN model assumes typical traffic and noise propagation conditions that are consistent with moderately adverse wind velocities and directions during the specified periods. The source of traffic noise is taken to be a line 0.5 m above the carriageway and 3.5 m from the nearside carriageway edge. The input variables for the CRTN model include traffic data, street configurations, road gradient, road surface nature, and absorbent ground cover. The traffic data include the total traffic volume, the traffic speed and the percentage of heavy vehicles on a road segment. Firstly, the basic noise level at a reference distance of 10 m away from the nearside carriageway edge is calculated based on hourly total traffic volume at a basic traffic velocity of 75 km/h, zero proportion of heavy vehicles, and zero road gradient. The corrections for traffic speed, the percentage of heavy vehicles, the road gradient, and the road surface nature are added to the basic noise level. Further corrections are needed to take into account the effects of propagation and reflection of traffic noise. The CRTN method determines the noise level exceeded for just 10% of the time over a period of one hour, *i.e.*, hourly *L*_10_. The simulated hourly *L*_10_ can be converted to the hourly equivalent sound level *L*_eq_. The conversion of *L*_10_ to *L*_eq_ has been mathematically described in [28]. In this study, *L*_eq_ is adopted to compare with the National Standard in China.

As the traffic noise model CRTN makes use of detailed information about the street configuration, manual collection of these complex spatial data from the Macao paper or digital maps is a tremendous task which takes time, incurs substantial labor expenditures, and in consequence, may lead to unintentional human errors. Therefore, a prototype urban landscape model has been programmed to integrate the CRTN model with ArcView GIS and hence, automate the noise modeling process. The spatial-related input parameters of the CRTN model such as traffic conditions, street configurations, road gradient, road surface nature, and absorbent ground cover percentage are extracted automatically by the urban landscape model from digital maps in ArcView format.

The cadastre map in ArcView is a polygon theme indicating the locations of land parcels. It is digitized from the 1:5000 paper cadastral map provided by the Macao Cartography and Cadastre Bureau. The road network map is created based on the 1:5000 paper cadastral map. In this study, manual traffic counts were conducted on major urban trunk roads and district distributors during the evening peak hour (18:00–19:00) on working days in 2010. On each studied road, the number of different vehicle categories passing was counted manually. Average vehicle speed on each studied road was determined according to manual records of vehicle passing times over a specified distance. The obtained traffic volumes of five categories of vehicle (*i.e.*, motorcycle, passenger car, taxi, truck and bus) and the traffic speed on each studied road have been stored in the attribute table of the road network map.

The green space map is a polygon theme which is used to determine the absorbent ground cover between the reception point and the edge of the nearside carriageway of the road for the CRTN model. The water coverage map is also a polygon theme which indicates the locations of lakes, reservoirs and lagoons in the Macao Peninsula. The green space map and the water coverage map are obtained from the Macao Cartography and Cadastre Bureau and will be used to provide urban land use data in different urban forms in the Macao Peninsula. The terrain map in ArcView is a surface theme which is used to provide the elevation of road surface. To create the terrain map for the study, a triangular irregular network (TIN) is first created for the Macao Peninsula by using the Arcview three-dimensional (3D) Analyst. A total of 2400 mass points with 3D coordinates x, y and z have been used as the vertices of the TIN. The 3D coordinates of the points are obtained from the Macao Cartography and Cadastre Bureau.

When the urban landscape model is executed, a target land parcel polygon is selected from the cadastral map in ArcView. Based on the coordinates of the vertices of the target land parcel polygon, the boundary segments of the land parcel polygon are obtained. If the boundary segment faces a road aligning parallel to it, a receptor point will be created in the middle of the sidewalk section between the boundary segment and the roadway. The traffic noise at the receptor point will represent the pedestrian exposure to traffic noise along the relevant sidewalk section.

The street configuration data (the length, width and orientation of the road), the traffic conditions (traffic volume, traffic composition and traffic speed), the absorbent ground cover percentage and the surface nature of the road segment in front of the receptor point are extracted and then input into the CRTN model to simulate the noise pollution at the target receptor point. The modeling hourly noise levels of *L*_10_ and *L*_eq_ are then stored in the attribute fields of the receptor point feature created in the receptor point map for pedestrian exposure to traffic noise. The spatial-related input variables are also stored in the attribute fields of the receptor point. When all the land parcels in the cadastral map are selected and manipulated, the urban landscape model stops the execution. All the modeling results can then be accessed from the receptor point map in ArcView. The statistical/spatial relationships of the input values and output results can also be investigated.

## 3. Spatial Distribution of Traffic Noise in the Macao Peninsula

**Figure 1.**
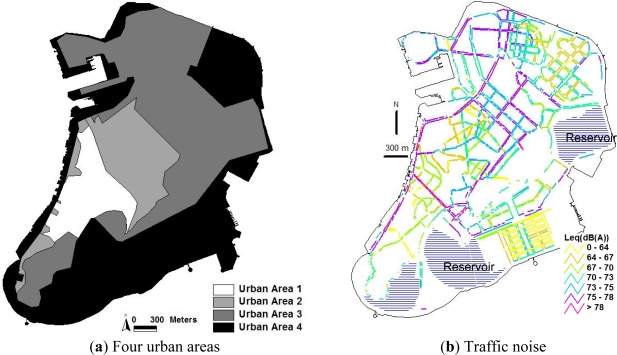
Spatial distribution of traffic noise in the four urban areas.

Pedestrian exposure to traffic noise in the Macao Peninsula is assessed in a traffic scenario using real traffic data. The traffic data including the total traffic volume, the traffic speed and the percentage of heavy vehicles on major urban trunk roads and district distributors have been obtained by manual traffic surveys conducted during the evening peak period (18:00–19:00) on working days in 2010. Figure 1 shows the spatial distribution of traffic noise levels modelled in the four urban areas of the Macao Peninsula. The four urban areas, namely Urban Areas 1, 2, 3, and 4, are divided according to four stages of urban growth in the Macao Peninsula, *i.e.*, 1557–1794, 1794–1912, 1912–1957, and 1957–present.

In this study, receptor points are created based on the land parcels in the cadastral map. As the sizes of the land parcels vary, the distribution of receptor points is not uniform. Consequently, the average traffic noise level in an urban area might be overestimated (or underestimated) if more receptor points are concentrated on a road with high (or low) traffic noise. To avoid errors induced by the non-uniform distribution of receptor points, the weighted average traffic noise level in each urban area is calculated instead. As the traffic noise at a receptor point represents the pedestrian exposure along the relevant sidewalk section, the length of the sidewalk section (*i.e.*, the length of the boundary segment of the land parcel) related to each receptor point is taken into account as a weight in statistical analysis. The weighted average traffic noise level in each urban area is calculated by a fraction, in which the numerator is the summation of weighted traffic noise values of all receptor points in the urban area and the denominator is the summation of the length of all the sidewalk sections related to these receptor points. The percentage of traffic noise levels exceeding the National Standard of 70 dB(A) is also calculated by a fraction, in which the numerator is the summation of the length of the sidewalk sections with the traffic noise above 70 dB(A) and the denominator is the summation of the length of all the sidewalk sections.

It is found that about 60% of traffic noise levels along the major pedestrian sidewalks in the Macao Peninsula in the evening peak hour (18:00–19:00) exceed the National Standard of 70 dB(A) in China (GB3096-2008). In particular, about 21% of traffic noise levels along the major pedestrian sidewalks are above the National Standard by 5 dB(A). In addition, the weighted average traffic noise levels (hourly *L*_eq_) in the Urban Areas 1, 2 and 3 are respectively 69.76 dB(A), 69.84 dB(A) and 69.3 dB(A), which are lower than the weighted average traffic noise levels of 72.76 dB(A) in the Urban Area 3. The spatial variance of traffic noise in the four urban areas will be analyzed in the following sub-sections.

### 3.1. Traffic Noise in the Urban Area 1

**Figure 2.**
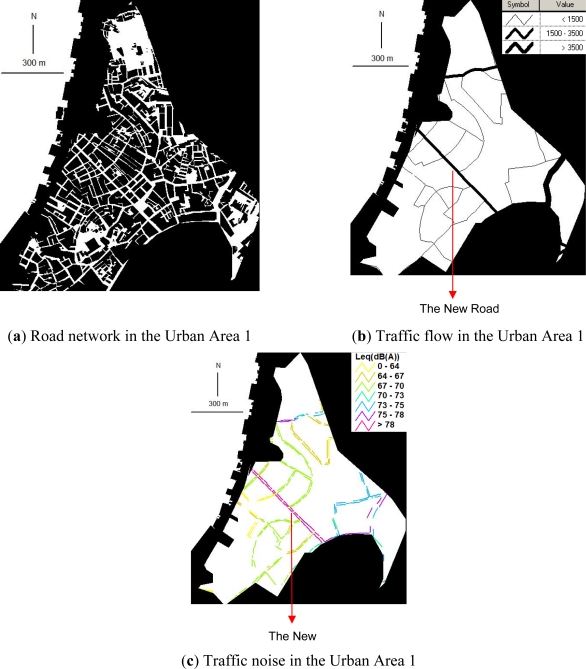
Spatial relationships of urban form, traffic flow and traffic noise in Urban Area 1.

Figure 2 shows the road network, traffic volume, and traffic noise in the Urban Area 1. In the Urban Area 1, the building lot space covers 71% of the urban area. While the road space, green space and water coverage cover only 8%, 2%, and 0%, respectively. Comparing with the other three urban areas, the Urban Area 1 has the most intensive land use as it has been developed since 1557. Besides residential houses, the Central Business District (CBD) lies in this area. The most famous historic landmarks are within walking distance of each other. In 2005, the historic centre of the Urban Area 1 was awarded ‘World Cultural Heritage’ status by the United Nations Educational, Scientific and Cultural Organization, citing the ‘dramatic mixing of eastern and western buildings in this jewel’ [29].

As shown in Figure 2(a), the road network is laid out in a complex curvature style which was originally designed for pedestrian transport and human-powered transport such as litters and sedan chairs. Study shows that about 65.8% of the roads in this historical urban area are less than 10 m wide. The complex curvature of the road network and the high percentage of narrow roads limit the traffic speed, traffic capacity, and parking capacity in the Urban Area 1. As shown in Figure 2(b), the traffic volumes on most roads are very low (around 200–400 vehicles/h) as drivers lack the motivation to drive vehicles into the area. As a result, about 65% of traffic noise levels along the major pedestrian sidewalks in this area does not exceed the National Standard of 70 dB(A), see Figure 2(c).

On the other hand, as shown in Figure 2(b), three straight roads cutting across the Urban Area 1 in the north, central and southeast part attract vehicle drivers to use as shortest paths to their destinations of the other urban areas. The great traffic demands (>1,500 vehicles/h) on the three straight roads lead to significantly higher traffic noise of 73–80 dB(A) than the other roads, as shown in Figure 2(c).

Particularly, the longest straight road (namely the New Road) passing through the heart of the Urban Area 1 was designed for vehicle use in the early 1900 s, which does not match the morphology of complex curved road network originally designed for pedestrian use during 1557–1794. Consequently, today’s drivers select the straight New Road rather than the other complex curved roads to pass through the heart of the Urban Area 1. It leads to a significantly higher traffic volume and hence, higher pedestrian exposure to traffic noise of above 78 dB(A) in the evening pear hour on the New Road. Noticeably, the high pedestrian exposure to traffic noise on the New Road reduces the comfort of tourists walking in the historic centre of the Urban Area 1 and is ruining the reputation of the area as a World Cultural Heritage site.

### 3.2. Traffic Noise in the Urban Area 2

**Figure 3.**
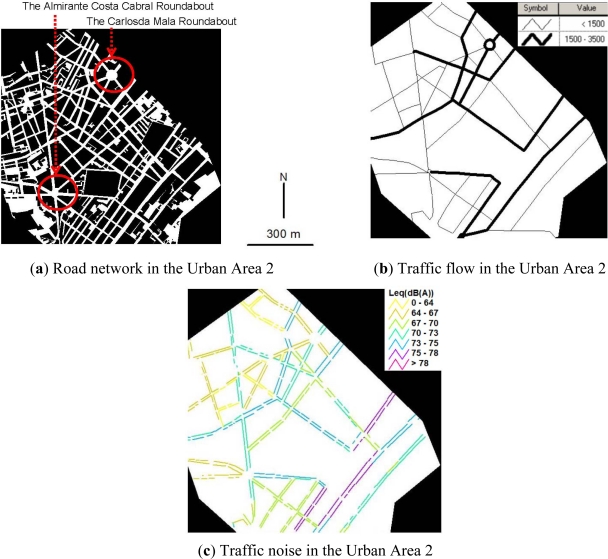
Spatial relationships of urban form, traffic flow and traffic noise in Urban Area 2.

Figure 3 shows the road network, traffic volume, and traffic noise in the Urban Area 2. Compared with the Urban Area 1, the building lot space in the Urban Area 2 has decreased to 54% and the road space has increased to 13%. However, there are still few of green space (3%) and water coverage (0%). The urban land use is still intensive in the Urban Area 2. Particularly, the land consumption per capita is only 13.2 m^2^ which is the lowest among four urban areas. The reason is that the urban land use of the Urban Area 2 is dominated by residential concerns and there are merely no commercial or industrial building lots in this urban area.

As shown in Figure 3(a), the roads in the Urban Area 2 radiate outwards from two roundabouts (*i.e*., the Carlosda Mala roundabout and the Almirante Costa Cabral roundabout). The radiated road network in the Urban Area 2 leads to higher traffic accessibility than the curved road network in the Urban Area 1. In addition, the proportion of roads 10–20 m wide has increased doubly from 20.5% in the Urban Area 1 to 40.6% in the Urban Area 2, which leads to higher traffic capacity in the Urban Area 2 than that in the Urban Area 1. The higher traffic accessibility and higher traffic capacity result in higher traffic volumes in the Urban Area 2. As observed in Figure 3(b), more than 50% of the roads in the Urban Area 2 have traffic volumes over 1,500 vehicles/h in the evening peak hour. About 50% of traffic noise levels along the major pedestrian sidewalks in the Urban Area 2 exceed the National Standard of 70 dB(A), see Figure 3(c).

Noticeably, two long straight roads in the southeast of the Urban Area 2 are part of the inner circumferential routes of the city and attract very high pass-through traffic. The two roads have traffic volumes higher than 2000 vehicles/h as shown in Figure 3(b). The great traffic demands on the two roads lead to significantly higher traffic noise of 73–76 dB(A) than the other roads, as seen in Figure 3(c).

### 3.3. Traffic Noise in the Urban Area 3

**Figure 4.**
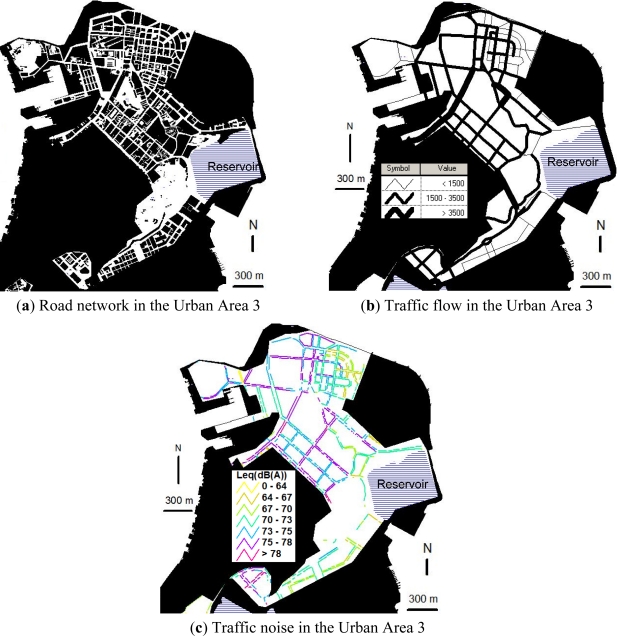
Spatial relationships of urban form, traffic flow and traffic noise in Urban Area 3.

Figure 4 shows the road network, traffic volume, and traffic noise in the Urban Area 3. The urban land use in the Urban Area 3 reflects modern urban design, which reserves more land for social welfare, industrial and transportation uses. Large areas of hills, which cover 11% of the Urban Area 3, are reserved for public recreation. Water coverage has increased to 10% as a result of construction of a reservoir for fresh water supply. Industrial zones are located in the north district where labor costs and land prices are lower. Flyovers have been built to reduce traffic congestion and to link with other urban areas.

As shown in Figure 4(a), the road network in the Urban Area 3 has a modern rectangular grid pattern. In addition, the proportion of roads wider than 20 m is 34.5% in the Urban Area 3, which is higher than the proportions of 13.7% and 10.7% in the Urban Areas 1 and 2, respectively. Due to the introduction of modern urban design such as the rectangular grid pattern of the road network and the increase in roads 20–40 m wide (boulevards designed with four lanes), the traffic capacity in the Urban Area 3 has considerably increased when compared to the Urban Areas 1 and 2. Besides, the flyovers in the Urban Area 3 have also reduced traffic congestion at road intersections and increased the traffic capacity. As a result, the majority of the roads in the Urban Area 3 have traffic volumes greater than 1,500 vehicles/h in the evening peak hour and in particular, urban trunk roads have traffic volumes greater than 3,500 vehicles/h, see Figure 4(b).

With the high traffic volumes on the majority of the roads, the Urban Area 3 has the highest traffic noise [weighted average L_eq_ of 72.76 dB(A)] among the four urban areas in the Macao Peninsula. As shown in Figure 4(c), the percentage of traffic noise levels along the major pedestrian sidewalks in the Urban Area 3 exceeding the National Standard of 70 dB(A) is about 81%, which is more than the percentages of 35% and 50% in the Urban Areas 1 and 2, respectively. Particularly, in the Urban Area 3, about 30% of traffic noise levels along the major pedestrian sidewalks in the evening peak hour exceed the National Standard by 5 dB(A).

### 3.4. Traffic Noise in the Urban Area 4

**Figure 5.**
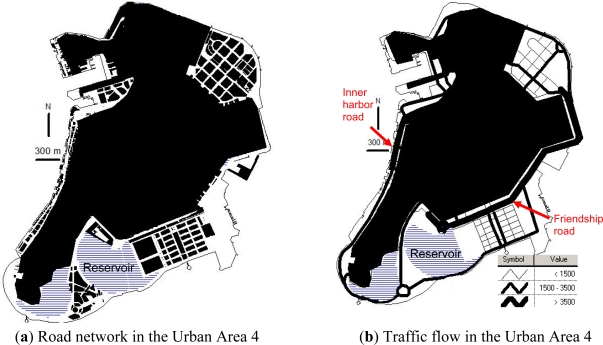

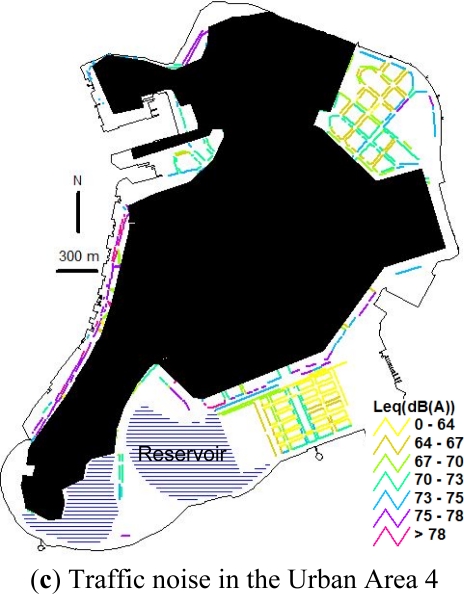
Spatial relationships of urban form, traffic flow and traffic noise in Urban Area 4.

Figure 5 shows the road network, traffic volume, and traffic noise in the Urban Area 4. The urban form of the Urban Area 4 is a result of cluster development. The building lot space only covers 11% of the Urban Area 4. While the road space, green space and water coverage cover 20%, 8%, and 24%, respectively. Comparing with the other three urban areas, the Urban Area 4 is less dense and the land consumption per capita reaches 53.9 m^2^. The water coverage increases significantly as a result of the reclamation of two lakes for preservation of the coastal landscape. Urban infrastructures such as the wastewater treatment plant and fishery/cargo harbors have been built in the northeast and west zones, respectively. Casinos, hotels, convention centers, commercial recreation facilities, commercial buildings and the ferry terminal are located along the reclaimed land belt in the south and east zones. There is still a large portion of undeveloped land in the south zone.

As shown in Figure 5(a), the road network in the Urban Area 4 is in the rectangular grid pattern to allow for wider roads and larger building blocks. The proportion of roads wider than 20 m is 84.8% in the Urban Area 4, which is significantly higher than the proportions of 13.7%, 10.7% and 34.5% in the Urban Areas 1, 2 and 3, respectively. The traffic congestion has been reduced by the adoption of more wider roads and a lower density of intersections in the road network design. In addition, the shortage of parking space has been resolved by forcing the high-rise residential and industrial buildings to reserve multi-storey car parks and by building more public outdoor and underground car parks near the casinos, convention centers, recreation facilities, hotels, ferry terminal and fishery/cargo harbors.

The fishery and cargo harbors induce greater logistic demands on the inner harbor road in the west zone, whereas the casinos, hotels, convention centers, commercial recreation facilities, commercial buildings and the ferry terminal induce greater passenger transport demands on the Friendship road, see Figure 5(b). Therefore, higher traffic volumes (>3,500 vehicles/h) in the evening peak hour are found on the two trunk roads (*i.e.*, the inner harbor road and the Friendship road) in the Urban Area 4. The distributors and circumferential routes connected to the two trunk roads also have higher traffic of around 1,500–2,000 vehicles/h. However, a number of roads with very low traffic of around 200–1,300 vehicles/h are found in the Urban Area 4. This can be explained by the implementation of blocked grid systems in the road network designs to ensure no major flow of through traffic in certain neighborhood areas, see Figure 5(b).

The high traffic volumes on the inner harbor road and the Friendship road lead to the high traffic noise (>73 dB(A)) on these two trunk roads, as shown in Figure 5(c). In particular, the traffic noise on the inner harbor road has reached 76–78 dB(A) in the evening peak hour. For the whole Urban Area 4, the weighted average traffic noise is 69.3 dB(A), which is the lowest in the four urban areas. Besides, the percentage of traffic noise levels along the major pedestrian sidewalks in the Urban Area 4 exceeding the National Standard of 70 dB(A) is 49%, which is more than the percentages of 35% in the Urban Area 1, but less than the percentage of 81% in the Urban Area 3.

The reason that the Urban Areas 1 and 2 have lower pedestrian exposure to traffic noise than the Urban Area 3 is because of lower traffic volumes in the Urban Areas 1 and 2. Comparing with the rectangular grid road networks and the high percentages of wider roads in the Urban Area 3, the complex curved or radiated road networks and the high percentages of narrow roads in the Urban Areas 1 and 2 limit the traffic capacity and lead to lower traffic volumes.

While the reason that the Urban Area 4 has the lowest pedestrian exposure to traffic noise in the four urban areas is mainly due to the implementation of blocked grid systems in the road network design which cause lower traffic in neighborhood areas in the Urban Area 4. In addition, the adoption of more wider roads and the increase of green space and water coverage also reduce traffic noise in the Urban Area 4. Wider roads lead to a longer propagation path and thus greater degradation of traffic noise to the receptors (*i.e.*, pedestrians) on the road; whereas larger green space and water coverage increase the absorbent ground cover percentage between the reception point and the edge of the nearside carriageway on the road and help absorbing traffic noise during propagation.

## 4. Conclusions

Macao Peninsula is a highly compact city due to its small area (9.3 km^2^), a high population density (49,763 inhabitants per km^2^) and a mixed land-use development (*i.e.*, a mixture of residential, commercial, industrial, or other land uses in a building or set of buildings). The high population density and the lack of vehicle limits (460 vehicles/km) have led to an increase in urban pollution. To provide useful information to local government and urban planners, this paper has investigated the spatial distribution of traffic noise in the Macao Peninsula. The interactions among urban form, traffic flow and traffic noise have been addressed. Considering the spatial nature of urban geometry and traffic, a high-resolution GIS-based traffic noise model system has been applied.

Pedestrian exposure to traffic noise in the Macao Peninsula has been assessed in a traffic scenario using real traffic data. Results indicate that the Macao Peninsula has fallen into a situation of serious traffic noise pollution. About 60% of traffic noise levels along the major pedestrian sidewalks in the evening peak hour exceed the National Standard of 70 dB(A) in China. In particular, about 21% of traffic noise levels along the major pedestrian sidewalks are above the National Standard by 5 dB(A).

The traffic noise levels in the four urban areas, namely Urban Areas 1, 2, 3 and 4, have been further analyzed. Each of the four urban areas has a unique urban form with different urban geometries and land use properties. Comparing with the rectangular grid road networks and the high percentages of wider roads in the modern Urban Area 3, the complex curved or radiated road networks and the high percentages of narrow roads in the historical Urban Areas 1 and 2 limit the traffic capacity and lead to lower traffic volumes. Therefore, the weighted average traffic noise levels in the historical Urban Areas 1 and 2 are respectively 69.76 dB(A) and 69.84 dB(A), which are lower than the weighted average traffic noise levels of 72.76 dB(A) in the modern Urban Area 3.

The Urban Area 4 has the lowest pedestrian exposure to traffic noise [69.3 dB(A)] in the four urban areas. The main reason is that the blocked grid systems implemented in the road network designs in the Urban Area 4 lead to lower traffic in neighborhood areas. In addition, the adoption of more wider roads and the increase of green space and water coverage also reduce traffic noise in the Urban Area 4.

Noticeably, the longest straight road (namely the New Road) passing through the heart of the historical Urban Area 1 has a significantly higher traffic volume and hence, higher pedestrian exposure to traffic noise of above 78 dB(A) during the evening peak hour. This has reduced the comfort of tourists walking in the historic centre of the Urban Area 1 and is ruining the reputation of the area as a World Cultural Heritage site. The local government should consider appropriate measures to control the traffic volume on the New Road.
